# Merosesquiterpene Congeners from the Australian Sponge *Hyrtios digitatus* as Potential Drug Leads for Atherosclerosis Disease

**DOI:** 10.3390/md15010006

**Published:** 2016-12-27

**Authors:** Huda A. Wahab, Ngoc B. Pham, Tengku S. Tengku Muhammad, John N. A. Hooper, Ronald J. Quinn

**Affiliations:** 1 Eskitis Institute for Drug Discovery, Griffith University, Brisbane 4111, Australia; nuruhuda.abdulwahab@griffithuni.edu.au or nhuda@umt.edu.my (H.A.W.); n.pham@griffith.edu.au (N.B.P.); john.hooper@qm.qld.gov.au (J.N.A.H.); 2 School of Fundamental Science, Universiti Malaysia Terengganu, Kuala Terengganu 21030, Malaysia; sifzizul@umt.edu.my; 3 Institute of Marine Biotechnology, Universiti Malaysia Terengganu, Kuala Terengganu 21030, Malaysia; 4 Queensland Museum, Brisbane 4101, Australia

**Keywords:** *Hyrtios digitatus*, merosesquiterpene, atherosclerosis, HepG2, SR-B1

## Abstract

A study of the chemical constituents from the Australian Sponge *Hyrtios digitatus* has provided a perspective on the connection between the chemistry and biology of the puupehenones, a unique and unusual class of merosesquiterpenes. In this study, a new tetracyclic merosesquiterpene, 19-methoxy-9,15-ene-puupehenol (**1**) was isolated from the marine sponge *Hyrtios digitatus* along with the known 20-methoxy-9,15-ene-puupehenol (**2**). Their structures were elucidated on the basis of spectroscopic data (^1^H and ^13^C NMR) in combination with experimental electronic circular dichroism (ECD) data. Compounds **1** and **2** are active at 1.78 μM and 3.05 μM, respectively, on Scavenger Receptor-Class B Type 1 HepG2 (SR-B1 HepG2) stable cell lines, targeting atherosclerosis disease.

## 1. Introduction

Marine diversity contributes a plethora of natural metabolites often without structural precedent elsewhere in the natural world [1,2]. During the past 30 years, more than 20,000 marine natural product compounds have been identified and marine derived compounds have been approved as therapeutics agents [3]. According to a recent review, 1378 new marine natural products were reported in the year 2014, indicating the marine environment as a huge source of chemical diversity and interestingly, 283 new compounds were identified from the phylum of Porifera [4]. Over the past 30 years, the FDA approved eight marine drugs which are Halaven^®^, Lovaza^®^, Adcetris^®^, Prialt^®^, Yondelis^®^, Cytosar-U^®^, Vira-A^®^, and Carragelose^®^ [5]. Currently, three marine compounds (plitidepsin, plinabulin, and tetrodotoxin) are in phase III clinical trials. The clinical trial pipeline for marine drugs is promising [6]. In the course of ongoing investigations aimed at the identification of compounds that can modulate atherosclerosis disease, we identified the Australian sponge *Hyrtios digitatus*, which showed anti-atherosclerotic activity in our initial screening studies, using a Scavenger Receptor-Class B Type 1 (SR-B1) reporter gene assay.

Marine sponges have generated a series of meroterpenes containing a terpenoid skeleton varying from sesqui-, di-, sester-, to triterpene units. The sesquiterpene quinones isolated from various species of marine sponges have biological activities such as antimicrobial [7], immunomodulatory [8], antileukemic [9], anti-malarial [10] and anti-HIV [11]. The genus *Hyrtios* (Demospongiae class, Dictyoceratida order, Thorectidae family) has proven to be a prolific producer of structurally diverse secondary metabolites [12]. It produces sesterterpenes [13,14], sesquiterpenes [15,16], macrolides [17,18], indoles, and β-carboline alkaloids, including puupehenone [19,20]. These compounds have diverse biological activities such as anti-cancer, anti-malaria, anti-neoplastic [21,22], and anti-angiogenic activities [23]. This is the first report from this genus on artherosclerosis studies.

**Figure 1:**
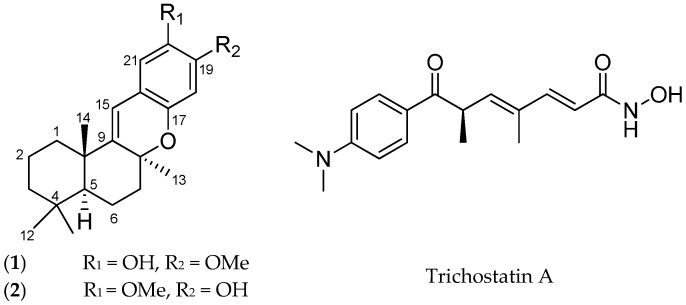
Chemical structures of compound **1**, **2**, and Trichostatin.

Herein, we report the isolation and structural elucidation of a new 19-methoxy-9,15-ene-puupehenol (**1**) and the known 20-methoxy-9,15-ene-puupehenol (**2**) (Figure 1) from an active fraction of the Australian sponge *Hyrtios digitatus*. Compound **2** was previously found from an Indo-Pacific *Hyrtios* sponge. The molecular structures of **1** and **2** were established on the basis of 1D and 2D NMR, UV, and HRESIMS data. The stereochemistry assignment of 1 and 2 using experimental and calculated electronic circular dichroism (ECD) is also described. Compounds **1** and **2** were evaluated for their up-regulatory activity on the anti-atherosclerotic SR-B1 HepG2 stable reporter cell line with the value of half maximal effective concentration (EC_50_) of 1.78 μM and 3.05 μM for **1** and **2**, respectively. Trichostatin A (TSA) was used as positive control displaying an EC_50_ of 5.25 μM.

## 2. Results and Discussion

The freeze-dried *Hyrtios digitatus* was sequentially extracted with *n*-hexane, dichloromethane (DCM), and methanol (MeOH). The DCM/MeOH extracts were combined and chromatographed using C18 bonded silica HPLC MeOH/H_2_O/0.1% trifluoroacetic acid (TFA) to yield a new 19-methoxy-9,15-ene-puupehenol (**1**) together with the known, 20-methoxy-9,15-ene-puupehenol (**2**), (Figure 1) [24]. 19-Methoxy-9,15-ene-puupehenol (**1**) was obtained as a pale yellow oil. The HRESIMS spectrum displayed a protonated molecular ion peak [M + H]^+^ at *m*/*z* 343.3418, which was consistent with the molecular formula C_22_H_30_O_3_. From the ^1^H and ^13^C NMR spectra, 30 proton and 22 carbon resonances were observed. The ^1^H NMR spectrum of **1** (see Figure S1) displayed a single methoxy proton signal (δ_H_ 3.68 ppm), four characteristic singlet methyl signals (δ_H_ 0.85, 0.92, 1.25, and 1.14 ppm), two aromatic protons (δ_H_ 6.22 and 6.67 ppm), five methylenes (δ_H_ 1.22, 1.49/1.60, 1.12/1.39, 1.64/1.76, and 1.88/2.01 ppm), one olefinic proton (δ_H_ 6.11 ppm) and one hydroxyl proton (δ_H_ 8.98 ppm) (Table 1).

**Figure 2:**
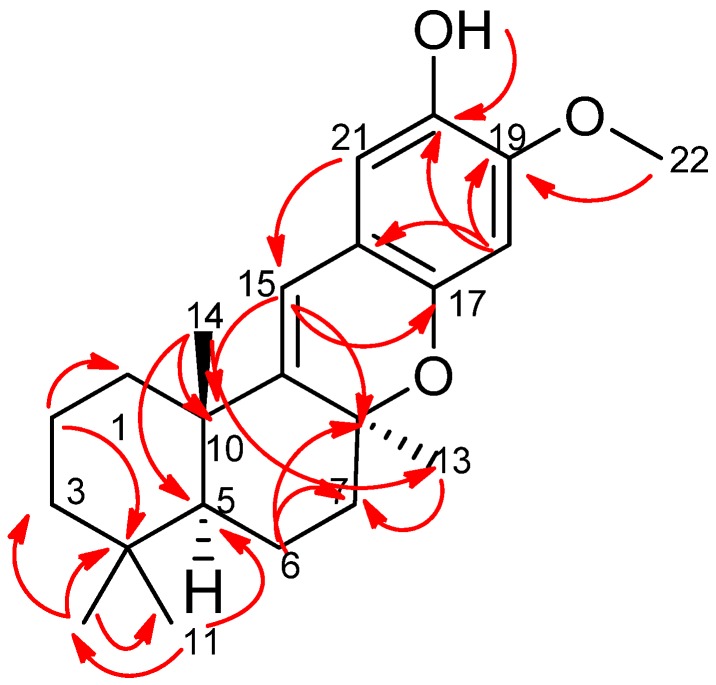
Key of heteronuclear multiple bond correlations (gHMBC) of **1**.

Further analysis of the ^13^C NMR and heteronuclear single quantum for proton-carbon correlation (gHSQC) spectra (Figure 2) indicated that compound **1** contained eight quaternary carbons (δ_C_ 32.6, 38.1, 75.9, 115.0, 141.9, 145.9, 148.7, and 146.8 ppm), two aromatic carbons (δ_C_ 103.9 and 110.9 ppm), one olefinic (δ_C_ 114.1 ppm), four methyl carbons (δ_C_ 21.6, 24.9, 25.2, and 33.4 ppm), one methine (δ_C_ 32.6 ppm), one methoxy (δ_C_ 56.7 ppm), and five methylenes (δ_C_ 16.7, 18.9, 30.6, 38.7, and 41.7 ppm). Analysis of correlation spectroscopy (gCOSY) and gHSQC data led to the assignment of the ABCD ring system in compound 1. A gCOSY spectrum displayed correlations from methylene proton H-6 (δ_H_ 1.64 ppm) to H-7 (δ_H_ 1.88 ppm), indicating the connectivity of C-6 (δ_C_ 16.7 ppm) and C-7 (δ_C_ 30.6 ppm).

Detailed heteronuclear multiple bond correlation (gHMBC) analysis showed that H-11 (δ_H_ 0.85 ppm) had gHMBC correlations with C-3 (δ_C_ 16.7 ppm), C-5 (δ_C_ 43.4 ppm), and C-12 (δ_C_ 16.7 ppm). gHMBC signals from proton H-12 (δ_H_ 0.92 ppm) to C-3, C-4 (δ_C_ 32.6 ppm), C-5, and C-11 (δ_C_ 33.4 ppm) suggested that H-11 and H-12 constituted a *gem*-dimethyl, connected at C-4 (δ_C_ 32.6 ppm). gHMBC correlations from H-2 to C-1 (δ_C_ 38.7 ppm), C-3 (δ_C_ 41.7 ppm), and C-4 (δ_C_ 32.6 ppm) and from H-14 (δ_H_ 1.14 ppm) to C-5 (δ_C_ 43.4 ppm) and C-10 (δ_C_ 38.1 ppm) completed the establishment of ring A. gHMBC analysis also indicated H-14 had correlations with C-9 (δ_C_ 148.7 ppm) and C-13 (δ_C_ 24.9 ppm), supporting the connection of rings A and B. A singlet methyl, H-13 (δ_H_ 1.25 ppm), displayed gHMBC correlations with C-7 (δ_C_ 30.6 ppm), C-8 (δ_C_ 75.9 ppm), and C-9 (δ_C_ 148.7 ppm). Two methylene signals H-6 and H-7 showed gHMBC correlations to carbons C-5, C-7, C-8, and C-6, C-14, respectively, and an olefinic proton signal H-15 (δ_H_ 6.11 ppm) had gHMBC correlations with C-8 (δ_C_ 75.9 ppm) and C-10 (δ_C_ 38.1 ppm), allowing for the connection of ring B and ring C.

**Figure 3:**
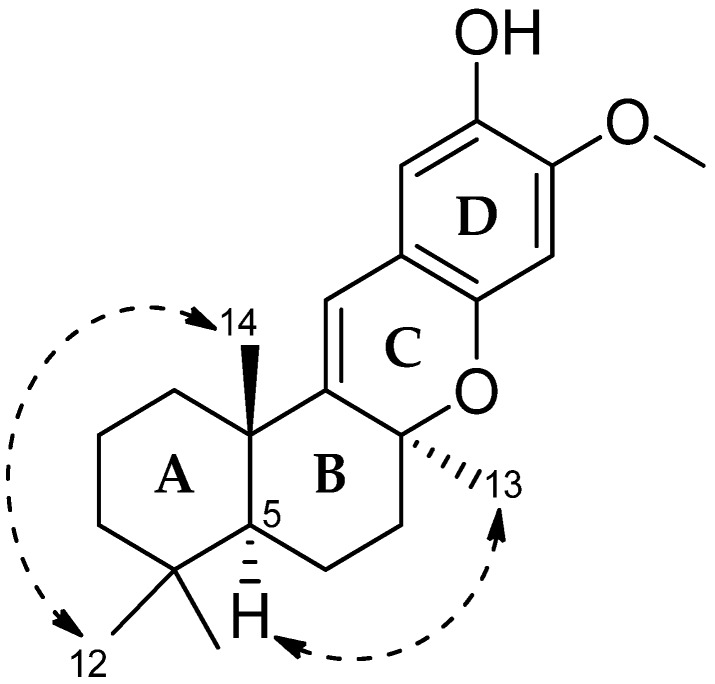
Selected nuclear overhauser effect spectroscopy (NOESY) correlations of **1**.

The establishment of ring C to ring D was confirmed by gHMBC correlations between the olefinic proton H-15 to C-16 (δ_C_ 115.0 ppm), C-17 (δ_C_ 145.9 ppm), and C-21 (δ_C_ 110.9 ppm). The methoxy proton signal H-22 (δ_H_ 3.68 ppm) was correlated with C-19 (δ_C_ 141.9 ppm), while a hydroxyl proton signal (δ_H_ 8.98 ppm) was correlated with C-18, C-19, and C-20. gHMBC correlations also observed from an aromatic proton signal, H-18 (δ_H_ 6.22 ppm) to C-17 (δ_C_ 145.9 ppm) and C-16 (δ_C_ 115.0 ppm), supported the C/D ring system. The relative stereochemistry of 1 was deduced on the basis of nuclear overhauser effect spectroscopy (NOESY) interactions (Figure 3). NOESY analysis disclosed interactions between two proton singlets, H-12 (δ_H_ 0.92 ppm) and H-14 (δ_H_ 1.14 ppm), which revealed their co-facial relationship and assignment as β-oriented. It indicated a *trans* fusion of rings A/B. Meanwhile, the NOESY correlation between a methine proton signal, H-5 (δ_H_ 1.37 ppm) and a methyl proton, H-13 (δ_H_ 1.25 ppm) implied a *cis* fusion of rings B/C. Taken together, the relative configuration of **1** was assigned as 5S*,8S*,10S* as shown in Figure 3.

The known compound, 20-methoxy-9,15-ene-puupehenol (**2**) was confirmed as C_22_H_30_O_3_ by accurate mass measurement and displayed a protonated molecular ion peak [M + H]^+^ at *m*/*z* 343.4532 in the (+)-HRESIMS spectrum [24]. A detailed comparison study of the NMR data of **1** indicated that **2** differed from **1** only at positions C-19 and C-20. NOESY was conducted to confirm the relative configuration as reported in the literature for the isolated compounds from the same genus Indo-Pacific *Hyrtios* sponge.

The absolute configuration of the known compound **2** had been obtained through degradative studies as 5*S*,8*S*,10*S* [24]. To confirm that **1** and **2** possess the same absolute configuration, ECD experimental and calculation studies by quantum mechanics were carried out. The OPLS-2005 force field in MacroModel was used to perform a conformational search with relative energies within 21 kJ/mol [25] and the basic set level (b3lyp/631gd) in solvent phase using the Gaussian 09 program, which was chosen for the geometric optimizations. There are three stereocenters with eight possible stereoisomers of **1**. All eight possible stereoisomers of **1** were subjected to the ECD calculation analysis. A conformational search using the OPLS-2005 force field in MacroModel gave the Boltzmann populations for (5*S*,8*S*,10*S*/5*R*,8*R*,10*R*)-**1**, (5*S*,8*R*,10*R*/5*R*,8*S*,10*S*)-**1**, (5*S*,8*R*,10*S*/5*R*,8*S*,10*R*)-**1**, and (5*S*,8*S*,10*R*/5*R*,8*R*,10*S*)-**1** as 98.8%, 95.6%, 99.2%, and 98.2%, respectively. The ECD spectra of four possible diastereomers were simulated at the (b3lyp/631gd) level in the solvent phase.

**Figure 4:**
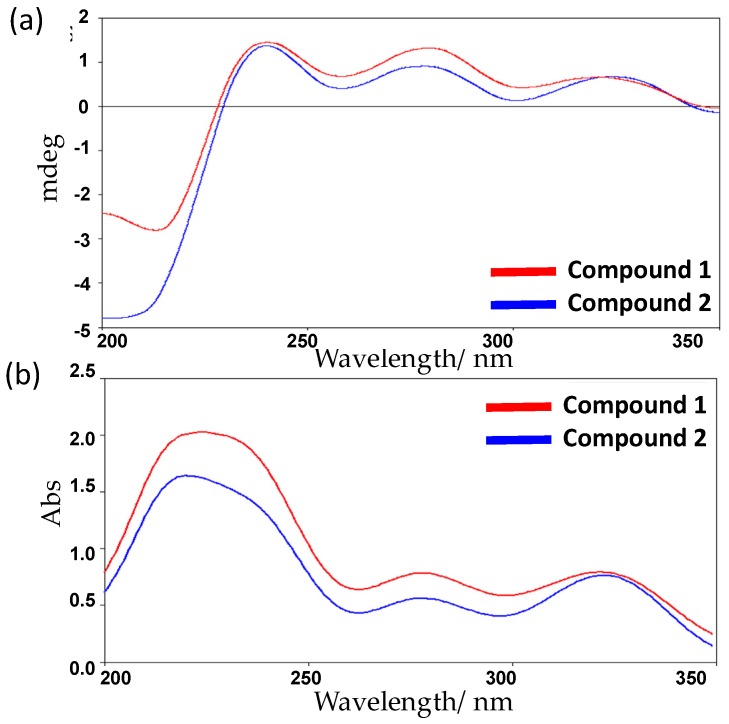
Experimental of an electronic circular dichroism (ECD) and ultraviolet (UV) spectra of **1** and **2**.

**Figure 5:**
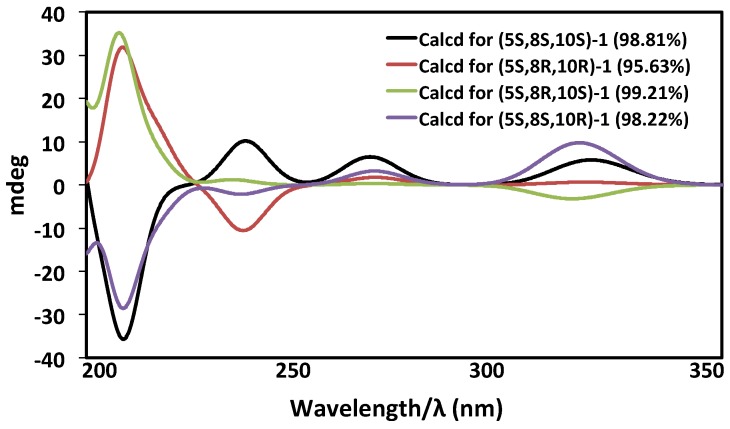
Comparison of the calculated ECD of four possible diastereomers of **1**.

**Figure 6:**
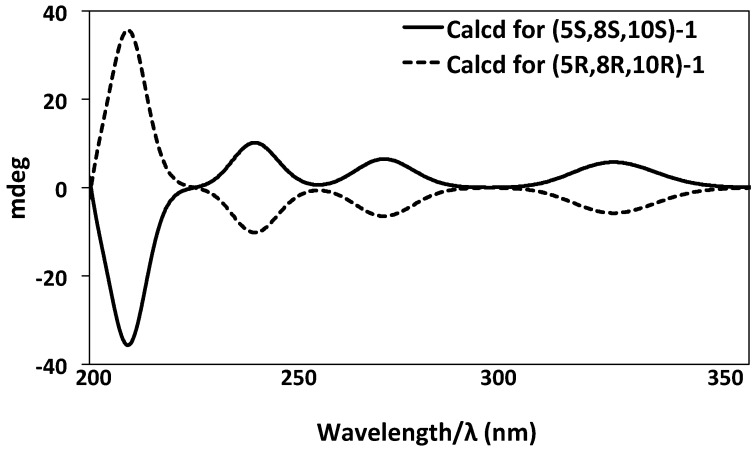
Comparison of the predominant conformer (5*S*,8*S*,10*S*)-**1** and (5*R*,8*R*,10*R*)-**1**.

The experimental ECD spectrum (Figure 4a), matched with the ECD spectrum of (5*S*,8*S*,10*S*)-**1** (Figure 5) with 98.8% relative populations. In the simulated ECD spectra of the four possible diastereomers of 1 as shown in Figure 5, the ECD spectrum of (5*S*,8*S*,10*S*)-**1** exhibited a clear designated ECD curve, with a negative peak at 214 nm and a positive peak at 238 nm, 278 nm, and 320 nm. The UV-Vis spectrum of **1** exhibited absorption maxima at 217, 235, 279, and 325 nm (Figure 4b), which agreed with the experimental ECD spectrum as in Figure 4a. Four bands at 217 nm for n→π* transition, 235 nm for π→π* transition, 279 nm for π→π* transition, and 325 nm for π→π* transition, were indeed easily observed in the UV-Vis spectrum of **1**. Thus, after considering the close similarities and supported by the ECD spectrum, the assignment of the absolute configuration of **1** as (5*S*,8*S*,10*S*) was confirmed (Figure 6). The spectrum of 1 was virtually identical to that of compound **2** (Figure 4a). Hence, compound **2** possesses the same absolute configuration for all stereogenic centers as compound **1**, (5*S*,8*S*,10*S*).

**Figure 7:**
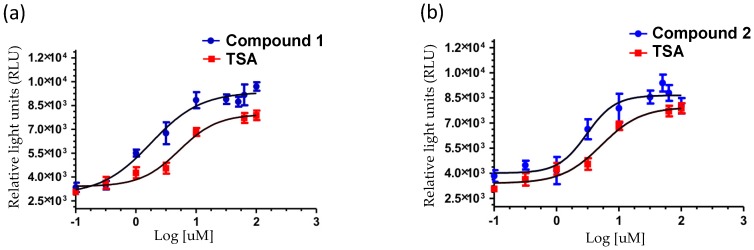
Dose-response curve of (**a**) Compound **1** and TSA; (**b**) Compound **2** and TSA.

Compounds **1** and **2** were evaluated for their up-regulatory activity on an SR-B1 HepG2 stable cell line using Trichostatin-A (TSA) as a positive control in a reporter gene assay. Both compounds increased the luciferase intensity of SR-B1 HepG2 cells in a dose-dependent manner with EC_50_ values for **1** and **2** of 1.78 μM and 3.05 μM, respectively (Figure 7). The efficacies of **1** and **2**, compared with TSA (100%), were estimated to be 130% and 121%, respectively (Table 2). Hence, compounds **1** and **2** are full agonists. It has been widely reported that SR-B1, the high-density lipoproteins (HDL) receptor, plays an important role in the development of atherosclerosis [26,27,28]. Zhang and his co-workers demonstrated an increase of 86% in mean atherosclerotic lesion of the proximal aorta in SR-B1^−/−^ as compared to SR-B1^+/+^ apolipoprotein E-deficient mice that strongly suggested the antiatherogenic nature of SR-B1 in an in vivo model [29]. Therefore, this study indicates the potential role of the merosesquiterpene class of compounds in reducing the progression of atherosclerosis. 

Drug-likeness is a property that is of importance for compounds in the drug discovery effort. There are various rules to evaluate the drug-likeness properties, such as Lipinski’s rule and Veber’s rule. Lipinski’s rule criteria states that molecular weight (MW) ≤500, partition coefficient (log P) ≤5, hydrogen bond acceptors (HBA) ≤10, and hydrogen bond donors (HBD) ≤5 [30]. In Veber’s rule, the other two parameters considered are that polar surface area (PSA) ≤140 and the number of rotatable bonds (NROT) ≤10 [31]. Thus, compound **1** and **2** were further investigated for drug-likeness evaluation using Lipinski’s rule and Veber’s rule.

The four Lipinski properties of compounds **1** and **2** were evaluated using the Instant *J*-Chem 5.12.0 software (Table 3). The results of the calculated MW, HBA, and HBD for compounds **1** and **2** comply with properties of Lipinski’s rule. Applying Veber’s rule (Instant *J*-Chem 5.12.0 software), compounds **1** and **2** demonstrated a PSA value of 38.69 and NROT as only 1 unit, satisfying two more requirements for drug-like molecules. Referring to the calculated value of Lipinski’s and Veber’s rule, compounds **1** and **2** have physicochemical properties consistent with predicted oral bioavailability [32] (Table 3). 

## 3. Materials and Methods

### 3.1. General

Optical rotations were measured on a JASCO P-1020 polarimeter (10 cm cell) (American Laboratory Trading, East Lyme, CT, USA). Circular dichroism spectra were measured on a JASCO J-715 Spectropolarimeter Circular Dichroism/Optical Rotatory Dispersion (JASCO, Easton, MD, USA). UV spectra were recorded on a CAMSPEC M501 UV/Vis spectrophotometer (CAMSPEC, Sawston, UK). NMR spectra were recorded at 30 °C on a 800 MHz spectrometer. The ^1^H and ^13^C chemical shifts were referenced to the DMSO-*d*_6_ solvent peaks at δ_H_ 2.50 and δ_C_ 39.52 ppm. Standard parameters were used for the 2D NMR spectra obtained, which included gCOSY, gHSQCA, gHMBCAD, NOESY, and ROESY. For HPLC isolation, a Waters 600 pump equipped with a Waters 966 PDA detector (Waters, Milford, MA, USA) and Gilson 715 liquid handler were used (Gilson, Lewis Center, Delaware County, OH, USA). An Onyx C18 column (5 μm, 21.2 mm × 150 mm) and Hypersil BDS C18 column (5 μm, 10 mm × 250 mm) were used for semi-preparative HPLC. A Phenomenex Luna C18 column (3 μm, 4.6 mm × 50 mm) was used for LCMS controlled by MassLynx 4.1 software (Waters, Milford, MA, USA). All solvents used for extraction and chromatography were HPLC grade purchased from RCI Labscan or Burdick & Jackson (Lomb Scientific, Sydney, Australia), and the H_2_O used was ultrapure water (Arium^®^ proVF, Sartorius Stedim Biotech, New York, NY, USA).

### 3.2. Animal Material

Samples of *Hyrtios digitatus* were collected at the depth of −17 m, latitude −21.70, longitude 152.55, Turner Reef, W. side, Swain Reefs, Queensland, Australia. It was identified as *Hyrtios digitatus* (phylum Porifera, class Demospongiae, order Dyctyoceratida, family Thorectidae). A voucher specimen G305703 has been deposited at the Queensland Museum, South Brisbane, Queensland, Australia.

### 3.3. Extractions and Isolation

The freeze-dried and ground sponge *Hyrtios digitatus* (5 g) was extracted exhaustively with *n*-hexane (250 mL), DCM (250 mL), and MeOH (2 × 250 mL), respectively. The DCM and MeOH extracts were combined and the solvent was evaporated to yield a dark brown residue (0.42 g). This extract was pre-adsorbed onto C18 (1 g) and packed dry into a small cartridge, which was connected to a C18 preparative HPLC column (5 μm, 21.2 mm × 150 mm). A linear gradient from 100% H_2_O (0.1% TFA) to 100% MeOH (0.1% TFA) was performed over 60 min at a flow rate of 9 mL/min and 60 fractions (1.0 min each) were collected. Pure 19-methoxy-9,15-ene-puupehenol (**1**) (2.36 mg, 0.046% dry weight) and 20-methoxy-9,15-ene-puupehenol (**2**) (2.12 mg, 0.042% dry weight) were obtained in fractions 31 and 33, respectively.

*(5S,8S,10S)-19-Methoxy-9,15-ene-puupehenol* (**1**): pale yellow oil; [α]${}_{D}^{25}$ +24.10° (*c* 0.2, MeOH), CD (MeOH) λmax (log ε) 238 (+3.98), 278 (+3.75), and 320 (+3.81) nm, UV (MeOH) λmax (log ε) 235 (+3.66), 279 (+3.75), and 325 (+3.81) nm, ^1^H (800 MHz, DMSO-*d*_6_) and ^13^C (150 MHz, DMSO-*d*_6_) NMR data are summarized in Table 1. Positive HRESIMS [M + H]^+^ at *m*/*z* 343.3418.

*(5S,8S,10S)-20-Methoxy-9,15-ene-puupehenol* (**2**): pale yellow oil; [α]${}_{D}^{25}$ +84.11° (*c* 0.2, MeOH), CD (MeOH) λmax (log ε) 242 (+3.68), 283 (+3.75), and 325 (+3.81) nm, UV (MeOH) λmax (log ε) 230 (+3.65), 277 (+3.74), and 322 (+3.81) nm, ^1^H (800 MHz, DMSO-*d*_6_) and ^13^C (150 MHz, DMSO-*d*_6_) NMR data are summarized in Table 1. Positive HRESIMS [M + H]^+^ at *m*/*z* 343.4532.

### 3.4. ECD Calculation

All molecular mechanics analyses and calculations were determined using Macromodel interfaced to the Maestro program [33,34]. The initial conformations search of 19-Methoxy-9,15-ene-puupehenol (**1**) and 20-Methoxy-9,15-ene-puupehenol (**2**) were optimized using the OPLS-2005 force field method applying a 21 kJ/mol energy window [25]. The optimized conformations were used for the ECD calculations, which were performed using the (b3lyp/631gd) basis set supported by Gaussian 09 software [35]. ECD spectra were generated using the program SpecDis Version 1.61 software (University of Wuerzburg, Wuerzburg, Germany) applying a Gaussian band shape with the width of 0.4 eV. Boltzmann distributions were estimated from the (b3lyp/631gd) free energies in the solvent model calculations.

### 3.5. Atherosclerosis Assays

Hepatocellular carcinoma cells (HepG2) stably transfected with SR-B1 were kindly provided by the Malaysian Institute of Pharmaceuticals and Nutraceuticals (IPharm), Penang, Malaysia. SR-B1 HepG2 stable cell lines were grown in MEM media (Life Technologies, Grand Island, NY, USA) supplemented with 10% fetal bovine serum (FBS). Cells were grown under 5% CO_2_ in a humidified environment at 37 °C. Thirty microliters (30 μL) of media containing 10,000 cells were added to a 384 well microtitre white clear bottom plate (Perkin Elmer, Waltham, MA, USA, part number: 353963) containing 0.3 μL of a compound. Final compound concentration range tested was 100 μM to 0.1 μM (final DMSO concentration of 1.0%). Each concentration in the media was tested in triplicate and in two independent experiments. Cells and compounds were then incubated for 48 h at 37 °C, 5% CO_2_ and 80% humidity. Cell proliferation was measured with the addition of 30 μL of a Luciferase reagent (Invitrogen, Carlsbad, CA, USA) solution to each well of the microtitre plate. The plates were incubated at room temperature for 10 min. The luminescence of each well was read on the VICTOR X Multilabel Plate Readers (Perkin Elmer). Nine-point concentration-response curves were then analyzed using non-linear regression and EC50 values were determined by using GraphPad Prism 5 (GraphPad Software Inc., La Jolla, CA, USA). Trichostatin-A (TSA) was used as a positive control compound.

## 4. Conclusions

Contributing to the chemical study on *Hyrtios digitatus*, a new (5*S*,8*S*,10*S*)-19-methoxy-9,15-ene-puupehenol (**1**) and the known (5*S*,8*S*,10*S*)-20-methoxy-9,15-ene-puupehenol (**2**) belonging to the merosesquiterpene structure class were identified. Structures of these compounds were determined by NMR spectroscopy and confirmed by ECD analysis. These two compounds satisfy properties for drug-like molecules with one violation (*c* logP) for oral bioavailability. This is the first time that the merosesquiterpene structure class has been reported to increase the activity of SR-B1 in a HepG2 cell line, indicating the potential role of these compounds in therapeutic intervention against atherosclerosis.

## Figures and Tables

**Table 1:** NMR data for **1** and **2** in DMSO-*d*_6_ *^a^*.

Position	1			2		
	δ_C_	δ_H_	gHMBC *^b^*	δ_C_	δ_H_	gHMBC *^b^*
1	38.7	1.22, m	C-2, 14	38.6	1.21, m	C-14
2	18.9	1.49, m 1.60, m	C-4, 10, 3 C-1, 3	18.6	1.49, m 1.60, m	C-4
3	41.7	1.12, m 1.39, m	C-2, 12, 11 C-2, 5	41.6	1.11, m 1.40, m	C-12, 5
4	32.6			33.4		
5	43.4	1.37, m	C-4, 6, 12, 14, 9, 10	43.5	1.38, m	C-6, 12, 14, 4, 10, 9
6	16.7	1.64, m 1.76, m	C-7, 4, 5, 8 C-7, 10, 5	16.8	1.65, m 1.76, m	C-4, 5 C-7, 10
7	30.6	1.88, m 2.04, m	C-6, 14, C-6, 14,	30.7	1.88, m 2.05, m	C-6, 13, 8, 5, 9 C-6, 13, 3, 8
8	75.9			75.9		
9	148.7			148.5		
10	38.1			38.1		
11	33.4	0.85, s	C-12, 3, 5	32.5	0.85, s	C-4, 12, 3
12	21.6	0.92, s	C-4, 3, 5, 11	21.1	0.92, s	C-4
13	24.9	1.25, s	C-7, 8, 9	24.9	1.25, s	C-7, 8, 9
14	25.2	1.14, m	C-1, 5, 8, 9, 10	25.4	1.14, m	C-10, 5, 9
15	114.1	6.11, s	C-10, 8, 21, 16, 17	114.0	6.11, s	C-14, 10, 8, 21, 16, 17
16	115.0			114.8		
17	145.9			145.5		
18	103.9	6.22, s	C-16, 20, 17, 19	103.9	6.22, s	C-16, 17, 20
19	141.9			146.6		
19-OH					8.95, s	C-18, 19, 20
20	146.8			141.9		
20-OH		8.98, s	C-18, 20, 19			
21	110.9	6.67, s	C-15, 20, 17, 19	111.0	6.67, s	C-15, 18, 19, 20
22	56.7	3.68, s	C-19	56.3	3.68, s	C-20

*^a^* 1H NMR at 800 MHz referenced to residual DMSO solvent (δ_H_ 2.50 ppm) and ^13^C NMR at 150 MHz referenced to residual DMSO solvent (δ_C_ 39.52 ppm); *^b^* gHMBC is referred to heteronuclear multiple bond correlation experiment.

**Table 2:** Effective concentration and % efficacy of **1**, **2**, and TSA.

Compound	EC_50_ (μM) ^b^	% Efficacy (E)
(5*S*,8*S*,10*S*)-19-methoxy-9,15-ene-puupehenol (**1**)	1.78	130
(5*S*,8*S*,10*S*)-20-methoxy-9,15-ene-puupehenol (**2**)	3.05	121
Trichostatin A ^a^	5.25	100

^a^ Positive control; ^b^ EC_50_ values are obtained from two independent experiments in triplicate.

**Table 3:** In silico physicochemical properties of **1** and **2** (in neutral forms).

Compound	MW	*c* logP	HBA	HBD	PSA	NROT	Predicted Bioavailability
**1**	342.34	5.05	3	1	38.69	1	√
**2**	342.45	5.05	3	1	38.69	1	√

## Supplementary Materials

The following are available online at www.mdpi.com/1660-3397/15/1/6/s1, Figure S1: ^1^H NMR Spectrum of **1** in DMSO-*d*_6_, Figure S2: ^13^C NMR Spectrum of **1** in DMSO-*d*_6_, Figure S3: ^1^H-^1^H COSY Spectrum of **1** in DMSO-*d*_6_, Figure S4: HSQC Spectrum of **1** in DMSO-*d*_6_, Figure S5: HMBC Spectrum of **1** in DMSO-*d*_6_, Figure S6: NOESY Spectrum of **1** in DMSO-*d*_6_, Figure S7: ROESY Spectrum of **1** in DMSO-*d*_6_, Figure S8: ^1^H NMR Spectrum of **2** in DMSO-*d*_6_, Figure S9: ^13^C NMR Spectrum of **2** in DMSO-*d*_6_, Figure S10: ^1^H-^1^H COSY Spectrum of **2** in DMSO-*d*_6_, Figure S11: HSQC Spectrum of **2** in DMSO-*d*_6_, Figure S12: HMBC Spectrum of **2** in DMSO-*d*_6_, Figure S13: NOESY Spectrum of **2** in DMSO-*d*_6_, Figure S14: ROESY Spectrum of **2** in DMSO-*d*_6_, Figure S15: Optimized structure of **1**, Figure S16: Optimized structure of **2**, Figure S17: Specimen Graphic, Table S1: Cartesian coordinate of **1** optimized: Standard orientation, Table S2: Cartesian coordinate of **2** optimized: Standard orientation.
