# Supplementary material for: Merosesquiterpene Congeners from the Australian Sponge Hyrtios digitatus as Potential Drug Leads for Atherosclerosis Disease
Source: Mar Drugs. 2016 Dec 27;15(1):6. doi: 10.3390/md15010006 (PMC5295226; doi:10.3390/md15010006)
Supplement: Supplementary file 1 [file marinedrugs-15-00006-s001.doc]

Supplementary Materials: Merosesquiterpene Congeners from the Australian Sponge
*Hyrtios digitatus* as Potential Drug Leads for Atherosclerosis Disease

Huda A. Wahab, Ngoc B. Pham, Tengku S. Tengku Muhammad, John N. A Hooper and
Ronald J. Quinn

**Figure S1.** ^1^H NMR Spectrum of **1** in DMSO-*d*_6_.

**Figure S2.** ^13^C NMR Spectrum of **1** in DMSO-*d*_6_.

**Figure S3.** ^1^H-^1^H COSY Spectrum of **1** in DMSO-*d*_6_*.*

**Figure S4.** HSQC Spectrum of **1** in DMSO-*d*_6_*.*

**Figure S5.** HMBC Spectrum of **1** in DMSO-*d*_6_.

**Figure S6.** NOESY Spectrum of **1** in DMSO-*d*_6_*.*

**Figure S7.** ROESY Spectrum of **1** in DMSO-*d*_6_.

**Figure S8.** ^1^H NMR Spectrum of **2** in DMSO-*d*_6_.

**Figure S9.** ^13^C NMR Spectrum of **2** in DMSO-*d*_6_.

**Figure S10.** ^1^H-^1^H COSY Spectrum of **2** in DMSO-*d*_6_*.*

**Figure S11.** HSQC Spectrum of **2** in DMSO-*d*_6_.

**Figure S12.** HMBC Spectrum of **2** in DMSO-*d*_6_*.*

**Figure S13.** NOESY Spectrum of **2** in DMSO-*d*_6_.

**Figure S14.** ROESY Spectrum of **2** in DMSO-*d*_6_.

**Figure S15.** Optimized structure of **1**.

**Table S1.** Cartesian coordinate of 1 optimized: Standard orientation.

| **Center Number** | **Atomic Number** | **Atomic Type** | **Coordinates (Angstroms)** | | |
| --- | --- | --- | --- | --- | --- |
|  |  |  | **X** | **Y** | **Z** |
| 1 | 8 | 0 | 5.258791 | 2.256495 | −0.43839 |
| 2 | 6 | 0 | 4.25379 | 1.34743 | −0.22664 |
| 3 | 6 | 0 | 2.931039 | 1.761853 | −0.22207 |
| 4 | 6 | 0 | 1.888706 | 0.844612 | −0.00924 |
| 5 | 6 | 0 | 0.484351 | 1.220256 | 0.120463 |
| 6 | 6 | 0 | −0.49273 | 0.29372 | 0.130965 |
| 7 | 6 | 0 | −1.96646 | 0.618516 | 0.46144 |
| 8 | 6 | 0 | −2.33685 | 2.009318 | −0.12135 |
| 9 | 6 | 0 | −3.84149 | 2.305178 | −0.09693 |
| 10 | 6 | 0 | −4.63177 | 1.226032 | −0.84375 |
| 11 | 6 | 0 | −4.41458 | −0.20495 | −0.29356 |
| 12 | 6 | 0 | −5.19414 | −0.38254 | 1.029137 |
| 13 | 6 | 0 | −5.03563 | −1.1879 | −1.31176 |
| 14 | 6 | 0 | −2.08863 | 0.674396 | 2.012366 |
| 15 | 6 | 0 | −2.86548 | −0.47319 | −0.22203 |
| 16 | 6 | 0 | −2.50476 | −1.91004 | 0.237069 |
| 17 | 6 | 0 | −1.01766 | −2.11293 | 0.609681 |
| 18 | 6 | 0 | −0.07602 | −1.15779 | −0.12483 |
| 19 | 6 | 0 | 0.041907 | −1.48621 | −1.62624 |
| 20 | 8 | 0 | 1.234238 | −1.41844 | 0.461289 |
| 21 | 6 | 0 | 2.216888 | −0.50368 | 0.194477 |
| 22 | 6 | 0 | 3.541747 | −0.93951 | 0.206031 |
| 23 | 6 | 0 | 4.558167 | −0.01051 | −0.00277 |
| 24 | 8 | 0 | 5.899451 | −0.28181 | −0.02507 |
| 25 | 6 | 0 | 6.328335 | −1.62193 | 0.192595 |
| 26 | 1 | 0 | 6.09833 | 1.761189 | −0.39767 |
| 27 | 1 | 0 | 2.711702 | 2.815841 | −0.37255 |
| 28 | 1 | 0 | 0.265367 | 2.275045 | 0.271764 |
| 29 | 1 | 0 | −1.98591 | 2.054257 | −1.16232 |
| 30 | 1 | 0 | −1.79913 | 2.795464 | 0.424588 |
| 31 | 1 | 0 | −4.20103 | 2.395662 | 0.937076 |
| 32 | 1 | 0 | −4.01937 | 3.283369 | −0.56436 |
| 33 | 1 | 0 | −4.32463 | 1.240638 | −1.90154 |
| 34 | 1 | 0 | −5.70672 | 1.457309 | −0.82959 |
| 35 | 1 | 0 | −6.26847 | −0.27355 | 0.828619 |
| 36 | 1 | 0 | −5.04803 | −1.37925 | 1.461159 |
| 37 | 1 | 0 | −4.93208 | 0.352057 | 1.793467 |
| 38 | 1 | 0 | −5.02634 | −2.22195 | −0.94836 |
| 39 | 1 | 0 | −6.08202 | −0.91626 | −1.50363 |
| 40 | 1 | 0 | −4.50089 | −1.15985 | −2.27077 |
| 41 | 1 | 0 | −1.25601 | 1.25544 | 2.424506 |
| 42 | 1 | 0 | −3.01483 | 1.159898 | 2.331585 |
| 43 | 1 | 0 | −2.06131 | −0.3179 | 2.473887 |
| 44 | 1 | 0 | −2.56901 | −0.40745 | −1.27989 |
| 45 | 1 | 0 | −2.77528 | −2.60578 | −0.56453 |
| 46 | 1 | 0 | −3.10953 | −2.20071 | 1.10313 |
| 47 | 1 | 0 | −0.7079 | −3.14512 | 0.403596 |
| 48 | 1 | 0 | −0.86236 | −1.95018 | 1.68252 |
| 49 | 1 | 0 | 0.418029 | −2.51067 | −1.7409 |
| 50 | 1 | 0 | 0.742188 | −0.79917 | −2.11434 |
| 51 | 1 | 0 | −0.91999 | −1.41169 | −2.14059 |
| 52 | 1 | 0 | 3.753393 | −1.98908 | 0.376757 |
| 53 | 1 | 0 | 6.024339 | −1.97723 | 1.185227 |
| 54 | 1 | 0 | 5.927646 | −2.29436 | −0.57632 |
| 55 | 1 | 0 | 7.417535 | −1.5968 | 0.128428 |

**Figure S16.** Optimized structure of **2**.

**Table S2.** Cartesian coordinate of **2** optimized: Standard orientation.

| **Center Number** | **Atomic Number** | **Atomic Type** | **Coordinates (Angstroms)** | | |
| --- | --- | --- | --- | --- | --- |
|  |  |  | **X** | **Y** | **Z** |
| 1 | 8 | 0 | 5.778816 | −1.43842 | 0.048204 |
| 2 | 6 | 0 | 4.545626 | −0.85563 | 0.068758 |
| 3 | 6 | 0 | 4.42441 | 0.538185 | −0.07505 |
| 4 | 8 | 0 | 5.621536 | 1.193216 | −0.22939 |
| 5 | 6 | 0 | 5.591806 | 2.603752 | −0.37976 |
| 6 | 6 | 0 | 3.158866 | 1.128781 | −0.0451 |
| 7 | 6 | 0 | 3.414247 | −1.65252 | 0.226696 |
| 8 | 6 | 0 | 2.153643 | −1.05613 | 0.239584 |
| 9 | 6 | 0 | 2.011664 | 0.335665 | 0.121003 |
| 10 | 6 | 0 | 0.665726 | 0.893571 | 0.2312 |
| 11 | 6 | 0 | −0.42335 | 0.104724 | 0.173326 |
| 12 | 6 | 0 | −1.85277 | 0.614086 | 0.460409 |
| 13 | 6 | 0 | −2.02175 | 2.045356 | −0.1156 |
| 14 | 6 | 0 | −3.47451 | 2.540243 | −0.11669 |
| 15 | 6 | 0 | −4.39315 | 1.574518 | −0.87437 |
| 16 | 6 | 0 | −4.3596 | 0.130186 | −0.32297 |
| 17 | 6 | 0 | −5.11811 | 0.061729 | 1.020891 |
| 18 | 6 | 0 | −5.11936 | −0.77194 | −1.32127 |
| 19 | 6 | 0 | −2.03449 | 0.664475 | 2.003562 |
| 20 | 6 | 0 | −2.86766 | −0.34101 | −0.25424 |
| 21 | 6 | 0 | −2.71492 | −1.81396 | 0.199368 |
| 22 | 6 | 0 | −1.27106 | −2.21857 | 0.576065 |
| 23 | 6 | 0 | −0.20377 | −1.38145 | −0.12841 |
| 24 | 6 | 0 | −0.16357 | −1.66742 | −1.64442 |
| 25 | 8 | 0 | 1.054278 | −1.85002 | 0.427256 |
| 26 | 1 | 0 | 6.430169 | −0.73014 | −0.07322 |
| 27 | 1 | 0 | 5.03086 | 2.899631 | −1.27419 |
| 28 | 1 | 0 | 5.162607 | 3.092807 | 0.50277 |
| 29 | 1 | 0 | 6.631265 | 2.917832 | −0.4908 |
| 30 | 1 | 0 | 3.041207 | 2.201902 | −0.13526 |
| 31 | 1 | 0 | 3.518017 | −2.72686 | 0.333991 |
| 32 | 1 | 0 | 0.584457 | 1.955924 | 0.440984 |
| 33 | 1 | 0 | −1.64393 | 2.059039 | −1.14616 |
| 34 | 1 | 0 | −1.4044 | 2.753364 | 0.449623 |
| 35 | 1 | 0 | −3.51357 | 3.531955 | −0.58391 |
| 36 | 1 | 0 | −3.83346 | 2.67812 | 0.909963 |
| 37 | 1 | 0 | −4.08602 | 1.557626 | −1.9296 |
| 38 | 1 | 0 | −5.42554 | 1.947102 | −0.86186 |
| 39 | 1 | 0 | −4.7157 | 0.736332 | 1.780092 |
| 40 | 1 | 0 | −6.16766 | 0.341707 | 0.868345 |
| 41 | 1 | 0 | −5.11363 | −0.94982 | 1.439774 |
| 42 | 1 | 0 | −5.22012 | −1.79986 | −0.95603 |
| 43 | 1 | 0 | −6.13134 | −0.38484 | −1.48731 |
| 44 | 1 | 0 | −4.61492 | −0.80971 | −2.29475 |
| 45 | 1 | 0 | −1.1569 | 1.119451 | 2.474543 |
| 46 | 1 | 0 | −2.89867 | 1.265851 | 2.299214 |
| 47 | 1 | 0 | −2.16342 | −0.32922 | 2.442589 |
| 48 | 1 | 0 | −2.55099 | −0.31251 | −1.30619 |
| 49 | 1 | 0 | −3.07078 | −2.4579 | −0.61087 |
| 50 | 1 | 0 | −3.36084 | −2.02741 | 1.056948 |
| 51 | 1 | 0 | −1.10603 | −3.27784 | 0.351273 |
| 52 | 1 | 0 | −1.11126 | −2.10969 | 1.654445 |
| 53 | 1 | 0 | 0.079124 | −2.72303 | −1.8104 |
| 54 | 1 | 0 | 0.603947 | −1.05779 | −2.13203 |
| 55 | 1 | 0 | −1.11704 | −1.46014 | −2.13857 |


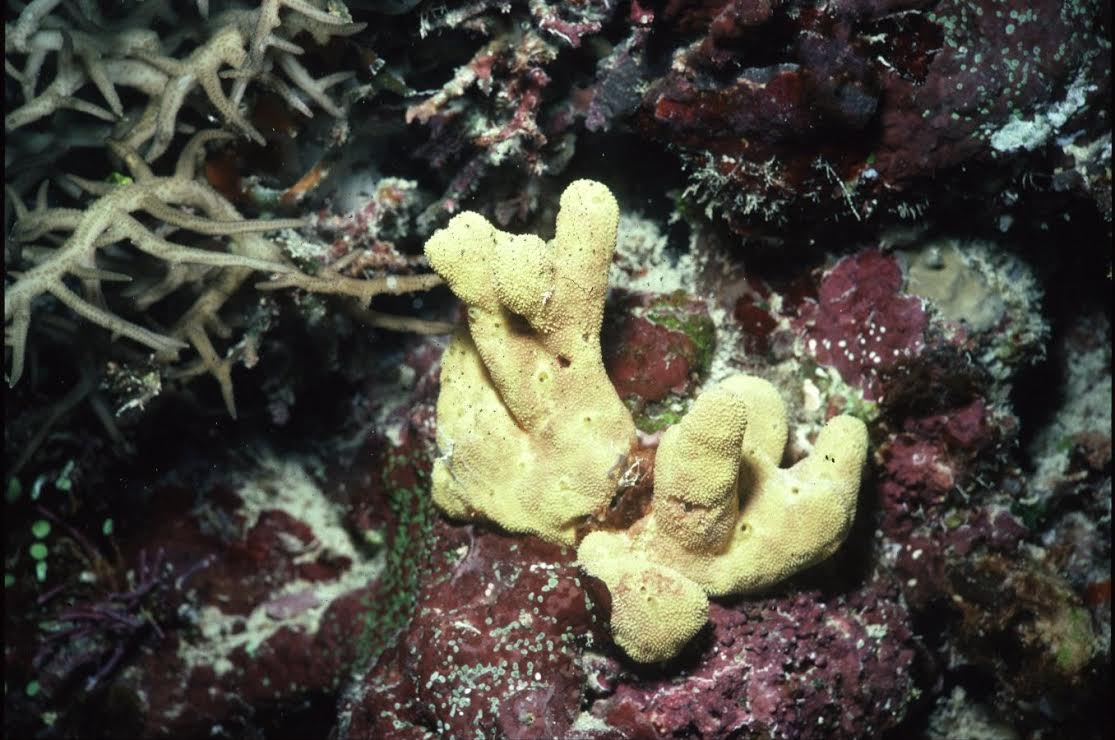


**Figure S17.** Specimen Graphic. Registration Number: Queensland Museum G305703. Locality: Turner Reef, W. side, Swain Reefs. Nation: Australia, Queensland. Latitude: −21.70138889. Longitude: 152.5505556. Depth: −17 m. Collection date: 28/07/1995. Habitat: bommies at entrance, back reef, sheer slope to 20 m, sand base. Collection method: SCUBA. Specimen identified by: JNA Hooper.
